# Luminal/extracellular domains of chimeric CI-M6PR-C proteins interfere with their retrograde endosome-to-TGN trafficking in the transient expression system

**DOI:** 10.7555/JBR.32.20180044

**Published:** 2018-06-20

**Authors:** Fei Chang, Na Li, Kang Yan, Yumin Huang, Hongfei Xu, Yongjian Liu

**Affiliations:** 1. Jiangsu Key Laboratory of Xenotransplanation, Department of Medical Genetics, Nanjing Medical University, Nanjing, Jiangsu 211166, China; 2. Department of Orthopedics, the First Affiliated Hospital of Nanjing Medical University, Nanjing, Jiangsu 210029, China; 3. Department of Pharmacology & Chemical Biology, University of Pittsburgh School of Medicine, Pittsburgh, PA 15213, USA.

**Keywords:** CI-M6PR, TGN targeting, retrograde trafficking, SNX5

## Abstract

The membrane trafficking of cation-independent mannose 6-phosphate receptor (CI-M6PR) between the *trans*-Golgi network (TGN) and endosomal compartments is not only critical for maintaining lysosomal function but also a well-known event for understanding molecular and cellular mechanisms in retrograde endosome-to-TGN trafficking. Although it has been well established in literature that the *C*-terminus of bovine CI-M6PR determines its retrograde trafficking, it remains unclear whether the luminal domain of the protein plays a role on these sorting events. In this study, we found that partial deletion of luminal domain of human CI-M6PR mistargeted the mutant protein to non-TGN compartments. Moreover, replacing the luminal domain of both bovine and human CI-M6PR with that from irrelevant membrane proteins such as CD8 or Tac also altered the TGN targeting of the chimeric proteins. On the other hand, only short sequence from HA fused with the transmembrane domain and *C*-terminus of the receptor, HA-hCI-M6PR-tail, resulted in its preferential targeting to TGN as for the full length receptor, strongly suggesting that sorting of the receptor may be influenced by luminal sequence. Furthermore, using this luminal truncated form of HA-hCI-M6PR as a model cargo, we found that the trafficking of the chimeric protein was regulated by the retromer complex through interacting with SNX5. In conclusion, our study strongly suggested that the disrupted luminal domain from hCI-M6PR or other irrelevant membrane proteins interfere with the process of membrane trafficking and TGN targeting of CI-M6PR.

## Introduction

The cation-independent mannose 6-phsophate receptor (CI-M6PR, also known as IGF-ⅡR) is known for its biological role in delivering lysosomal hydrolases to endosomes for maintaining the normal function of lysosome and cellular homeostasis^[1]^. To ensure these functions, the receptor requires efficient trafficking from the *trans*-Golgi network (TGN) with ligand M6P to the endosomal pathway for discharging its ligand and then recycling back to TGN to be reused^[2^‒^4]^. Over the past decades, this highly regulated subcellular membrane trafficking event has also been the main focus for understanding molecular mechanisms underlying endosome-to-TGN retrograde trafficking^[3^,^5]^. CI-M6PR is a classic type I membrane protein with a luminal/extracellular domain for ligand binding, a transmembrane domain (TMD) and a cytoplasmic domain^[3]^. Specifically, the cytoplasmic domain of the receptor was characterized for several sorting signals in determining its TGN-targeting and subcellular trafficking^[3]^. For example, by binding to cytosolic sorting machinery AP1, GGAs, and the phosphofurin acid cluster sorting protein-1 (PACS-1), its acidic-cluster-dileucine (AC-LL) signal plays an important role in the sorting of CI-M6PR^[6^‒^9]^. Additionally, this domain also contains a binding site for the tail-interacting protein 47 (TIP47) which is involved in its retrograde recycling from the endosome to the TGN^[10^‒^11]^. Interestingly, these molecular mechanisms were revealed to be heavily based on some of the previous established cell model systems such as using a CD8 chimeric protein in which the cytoplasmic domain of CD8 was used to replace the cytoplasmic domain of bovine CI-M6PR^[12^‒^14]^.

Recently, we have attempted to create a human CI-M6PR chimeric construct using the cytoplasmic domain of human CI-M6PR which is highly similar to that of bovine (76% sequence similarity), assuming identical subcellular localization and trafficking mechanisms. However, our study indicated that CD8-hCI-M6PR-C failed to efficiently target to the TGN compartments. Thus, we started to dissect the human CI-M6PR to determine which domains are required for its TGN targeting and trafficking. The luminal domain of the human CI-M6PR has a repetitive structure that consists of 15 repeats of approximately 174 amino acids for each^[1]^. Containing two distinct M6P-binding sites (repeating segments 3 and 9) and a single IGF-Ⅱ binding site (segment 11), this domain seemed to be unique for its ligand binding^[15^‒^16]^. Nevertheless, the role of the luminal domain in regulating membrane trafficking of the receptor has not been well characterized.

Here we report that efficient TGN targeting of both bovine and human CI-M6PR can be disrupted by partial luminal domain of the receptor. Furthermore, the chimeric proteins containing the luminal and transmembrane domains of two irrelevant membrane proteins and *C*-terminus of CI-M6PR also showed altered non-TGN localization. We have proposed a novel model system, HA-hCI-M6PR-tail, for studying retrograde trafficking of TGN preferential targeted cargo protein. In addition, our data confirmed that the retrograde trafficking of the tagged CI-M6PR was regulated by the retromer component SNX5.

## Materials and methods

### Reagents and antibodies

Cycloheximide (CHX) and poly-D-lysine was purchased from Sigma Chemical Co (St Louis, MO, USA). Matrigel was purchased from Becton Dickinson (Franklin Lakes, NJ, USA). The following reagents were purchased from Thermo Fisher (Waltham, MA): Protein A/G Agarose, Dulbecco's Modified Eagle Medium (DMEM), Lipofectamine 2000 and penicillin/streptomycin. Cosmic calf serum (CCS) was from HyClone (GE Life Science, Marlborough, MA, USA). All the restriction enzymes and products related to plasmid constructions were from NEB (Ipswich, MA, USA). The following primary antibodies were used: mouse monoclonal anti-HA (Covance, Princeton, NJ, USA), rabbit polyclonal anti-HA (Covance, Princeton, NJ, USA), mouse monoclonal anti-Flag (Sigma), mouse monoclonal anti-CD222 and sheep polyclonal TGN46 (Bio-Rad, Hercules, CA, USA), mouse monoclonal LAMP1, Golgin97 and EEA1 (Thermo Fisher), and mouse monoclonal anti-Myc (Santa Cruz Biotechnology, Santa Cruz, CA, USA). The following secondary antibodies from Thermo Fisher were used: Alexa 488-conjugated donkey anti-mouse secondary antibody, Alexa488-conjugated donkey anti-rabbit secondary antibody, Alex 488-conjugated donkey anti-sheep secondary antibody, Alexa568-conjugated donkey anti-mouse secondary antibody, Alexa568-conjugated donkey anti-rabbit secondary antibody, HRP-conjugated goat anti-mouse secondary antibody, and HRP-conjugated goat anti-rabbit secondary antibody.

### DNA constructs

pCMV5-Myc-hCI-M6PR was a gift from Dr. Richard G. MacDonald (University of Nebraska Medical Center, USA) and confirmed by sequencing analysis based on Gene ID NM_000876. The DNA sequence of cytoplasmic domain of bovine CI-M6PR was synthesized from GenScript (Piscataway, NJ, USA) and further confirmed by sequencing analysis based on Gene ID J03527.1. To construct hCI-M6PR-tail and hCI-M6PR-△D1-13, the DNA fragments were amplified from pCMV5-Myc-hCI-M6PR by PCR using a pair of primers containing *Hind*Ⅲ and *Xho*Ⅰ sites for subcloning into pcDNA3.1-3HA vector. cDNA for human CD8 was a gift from Dr. Tuanlao Wang (Xiamen University, China) and was confirmed by sequencing analysis based on Gene ID NM_001768.6. Tac was used in our previous paper based on Gene ID NM_000417.2^[17]^. Overlap extension PCR was used to construct chimeric proteins. Taking CD8-CI-M6PR-C for example, the DNA sequences containing the luminal domain and TMD of CD8, and the cytoplasmic domain of hCI-M6PR were amplified by PCR separately using specific primers followed by subcloning into pcDNA3.1-3HA vector. CD8-hCI-M6PR-tail-LD was created based on the CD8-CI-M6PR-C. All the primers used in the study were shown in ***Supplementary Table 1***, available online.

### Cell culture and transfection

HeLa Swiss and COS7 cells were cultured in DMEM supplemented with 10% CCS and 1% penicillin/streptomycin at 37 °C and 5% CO_2_. Transient transfection and expression were performed by transfecting the plasmids using Lipofectamine 2000, according to the manufacturer's instructions. The transfected cells were harvested 24‒48 hours later for analysis.

### Immunofluorescence and confocal microscopy

HeLa Swiss cells were seeded on poly-D-lysine and Matrigel-coated glass coverslips 24 hours before transfection followed by transfection using Lipofectamine 2000 according to the manufacturer’s instructions. 24 hours later, cells were fixed with 4% paraformaldehyde followed by permeabilization and blocking for 30 minutes in blocking solution (2% BSA, 1% fish skin gelatin and 0.02% saponin in PBS). Cells were then incubated with primary antibody in blocking solution for 1 hour and followed by incubation with the appropriate Alexa 488 or 588-conjugated secondary antibody for 1 hour. For confocal laser microscopy, staining was visualized with a confocal laser microscope (LSM710, Zeiss) and the images processed using the NIH Image program and ZEN program.

### Antibody uptake assay

The uptake assay was performed as described previously^[18]^. HeLa Swiss cells expressing the HA-hCI-M6PR-tail constructs were grown to 70% confluency on 13-mm coverslips. After washing the cells with ice-cold PBS, the coverslips were incubated in chilled media for 15 minutes at 4 °C making sure that all trafficking had stopped at the time of the antibody incubations. The cover slips were then washed again in ice-cold PBS, blotted dry, and then incubated with 100μL DMEM containing anti-HA antibody for 30 minutes at 4 °C before being washed again with cold PBS and then transferred to wells containing prewarmed media at 37 °C. After incubation for 0, 8, 16, or 24 minutes, the coverslips were washed and fixed with 4% paraformaldehyde. The continuous uptake assay was performed in a similar procedure as immunofluorescence staining.

### Co-immunoprecipitation and Western blot analysis

COS7 cells transfected with different expression constructs were lysed in NP-40 lysis buffer (150 mmol/L NaCl, 50 mmol/L Tris-HCl, pH 8.0, 5 mmol/L EDTA, 0.5% NP-40, 1 mmol/L PMSF and 0.1 mmol/L leupeptin) for 10 minutes on ice. Cell lysate was centrifuged at 1,600 *g* and the supernatants were then precleared by incubation for 60 minutes at 4 °C with 30μL protein A/G agarose beads and centrifugation at 8,000 *g* for 5 minutes. The precleared lysates were incubated for 2 hours at 4 °C with 30μL protein A/G agarose beads bound to polyclonal antibody to tagged protein. After immunoprecipitation, the beads were washed in wash buffer (0.5% NP-40, 150 mmol/L NaCl, 50 mmol/L Tris-HCl, pH 7.0 and 5 mmol/L EDTA) and then processed for SDS-PAGE analysis. For Western blotting, proteins in sample buffer were separated by electrophoresis through discontinuous 10% SDS-polyacrylamide gels before electrotransfer to nitrocellulose (Tanon, Shanghai). The filters were then blocked in PBS containing 0.1% Tween-20 (TBS) and 5% nonfat dry milk, incubated in TBS with 1% nonfat dry milk and primary antibody at dilutions from 1:1,000 to 10,000, washed in TBS, and incubated in appropriate secondary antibody conjugated to peroxidase followed by washing in TBS and visualization by enhanced chemiluminescence with the Tanon 5200 gel imaging system (Tanon, Shanghai).

### Protein half-life analysis

HeLa Swiss cells were transfected with different expression constructs using Lipofactamine 2000 in 12-well plates. After 24 hours of transfection, cells were treated with 100μg/mL CHX which is used to block the protein synthesis and samples were then collected at the time interval of 0, 6, or 12 hours. Cells were lysed and subjected to Western blot analysis.

### Statistical analyses

Statistical analysis was performed using Origin 8.0. The expression level in half-life studies were quantified by densitometry of the bands between two treatment groups and statistical significance were performed by one-way ANOVA followed by Turkey’s post hoc test, and denoted * if *P*<0.05, ** if *P*<0.01 and *** if *P*<0.001. Results were expressed as mean±SEM if not indicated otherwise.

## Results

### The chimeric protein of CI-M6PR failed to target to the TGN

**Fig.1:**
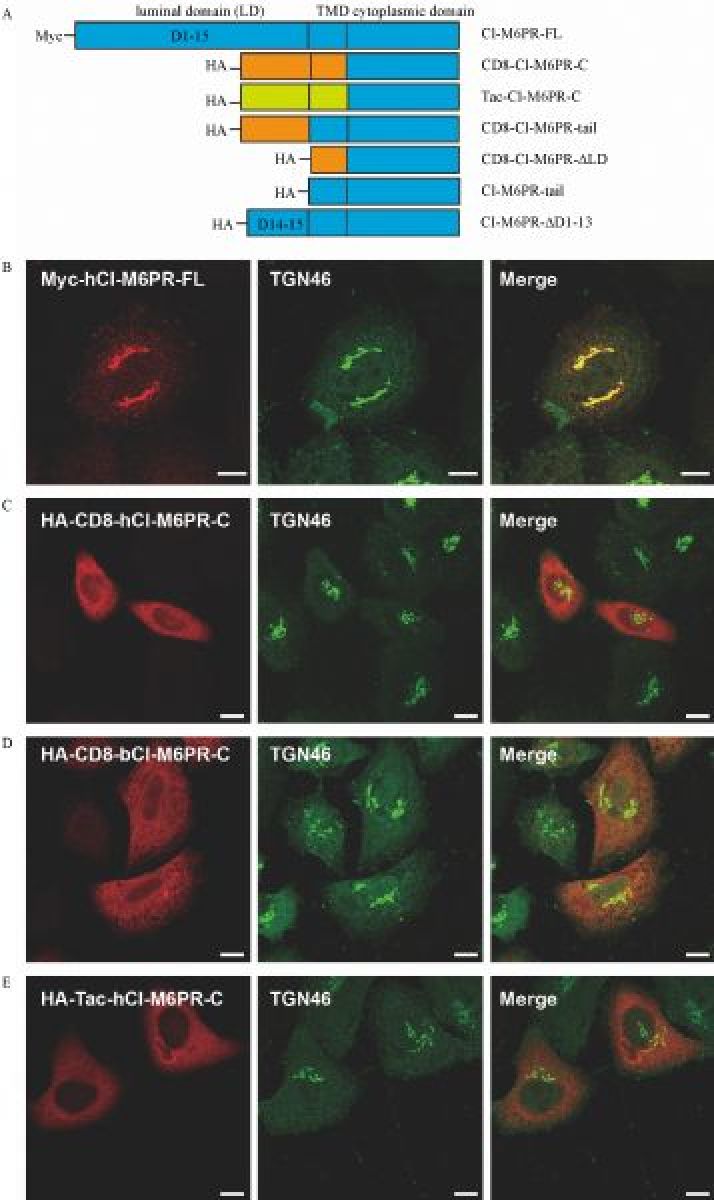
**The chimeric protein of CI-M6PR failed to target to the *trans*-Golgi network (TGN). ** A: Schematic representation of different CI-M6PR truncated or chimeric proteins used in this study. Tag is attached to the N-terminus of each construct to avoid the possibility of disturbing the sorting signals in its *C*-terminus. Sequences from different proteins are color coded. TMD: transmembrane domain. B–E: The co-localization of TGN46 with Myc-hCI-M6PR-FL (B), HA-CD8-hCI-M6PR-C (C), HA-CD8-bCI-M6PR-C (D), and HA-Tac-hCI-M6PR-C (E) was examined by double immunofluorescent staining in HeLa Swiss cells transiently transfected with specific constructs. Scale bar, 10 μm.

Our current understanding about TGN targeting of CI-M6PR was mainly based on the analysis of the decisive role of the *C-*terminus of bovine CI-M6PR in its trafficking. We intended to study membrane trafficking of human CI-M6PR and assumed that its *C*-terminus would carry similar TGN targeting signals as the amino acid sequences of this region between bovine and humans are highly similar (76% similarity, data did not show). More importantly, the well-established sorting motifs within this region of bovine CI-M6PR are conserved in human sequence. We first constructed a chimeric protein by fusing the cytoplasmic domain of the human CI-M6PR with the luminal and transmembrane domain of CD8, or CD8-hCI-M6PR-C, based on the similar strategy using bovine CI-M6PR^[2]^ (***Fig. 1A***). As shown in ***Fig. 1B***, the subcellular localization of transiently expressed Myc-hCI-M6PR-FL in Hela Swiss cells was clearly localized to TGN compartments compared with the marker protein TGN46. Surprisingly, immunostaining analysis showed that the majority of overexpressed chimeric proteins HA-CD8-hCI-M6PR-C were not significantly co-localized with TGN46 (***Fig. 1C***). This data is inconsistent with the previous work when the similar chimeric protein containing bovine *C*-terminus of CI-M6PR was stably overexpressed in Hela M cells which was preferentially TGN localized^[2]^. For a simple verification, we then used the synthesized DNA sequences of the bovine CI-M6PR *C*-terminus to construct HA-CD8-bCI-M6PR-C (***Fig. 1A***) following the same strategy as reported^[2]^. Upon overexpression in HeLa Swiss cells, the chimeric CD8-bCI-M6PR-C showed a non-TGN localization pattern, similar to that with CD8-hCI-M6PR-C (***Fig. 1D***). These results indicated that the luminal/extracellular domain and TMD from CD8 introduced to the chimeric proteins might alter their TGN targeting. To further confirm this possibility, we created a new chimeric protein fused the cytoplasmic domain of hCI-M6PR with another irrelevant membrane protein Tac (***Fig. 1A***). As shown in ***Fig. 1E***, overexpressed Tac-hCI-M6PR-C also showed the peripheral non-TGN localization pattern subcellularly as they were poorly co-localized with TGN46.

### The luminal domains of the chimeric proteins influence TGN-targeting

**Fig.2:**
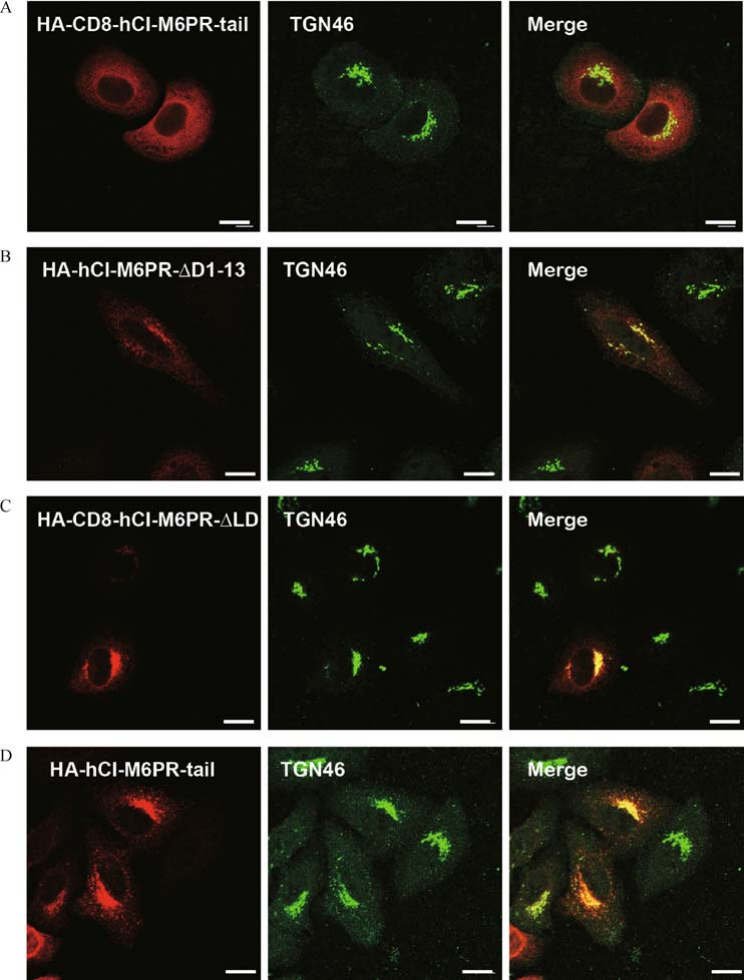
**The luminal domain of the chimeric proteins influence TGN-targeting.** A–C: The co-localization of TGN46 with HA-CD8-hCIM6PR-tail (A), HA-hCI-M6PR-ΔD1-13 (B), HA-CD8-hCI-M6PR-ΔLD (C), and HA-hCI-M6PR-tail (D) was examined by double immunofluorescent staining in HeLa Swiss cells transiently transfected with specific constructs. Scale bar, 10 μm.

To investigate whether the TMD of CI-M6PR contributes to its normal localization, we created HA-CD8-hCI-M6PR-tail by replacing the TMD of CD8 with that of hCI-M6PR in HA-CD8-hCI-M6PR-C construct (***Fig. 1A***). The immunostaining on the overexpressed chimeric protein in Hela Swiss cells showed that HA-CD8-hCI-M6PR-tail had a similar distribution pattern with that of HA-CD8-hCI-M6PR-C, with no remarkable co-localization with TGN46, excluding a role of the TMD of CI-M6PR in determining its TGN-targeting (***Fig. 2A***). This result strongly indicated that the chimeric luminal domain may contribute to the unusual distribution of these chimeric proteins. To determine whether the luminal domain of human CI-M6PR participates in TGN sorting, we constructed a deletion mutant of hCI-M6PR by removing the first 13 repeating segments (1‒13) within its luminal domain (***Fig. 1A***). As shown in ***Fig. 2B***, the deletion mutant showed significantly reduced co-localization with TGN46, confirming that the partial luminal domain may play a negative role in determining the protein’s TGN-location. Finally, by replacing the luminal domain with short triple HA sequences, we made constructs only containing the cytoplasmic domain of CI-M6PR and TMD from either CD8 or CI-M6PR. The subcellular localization analysis on these chimeric proteins showed clear perinuclear TGN staining pattern based on their significant co-localization with TGN46 (***Fig. 2C***&***D***). In conclusion, the partial deletion of luminal domain or irrelevant luminal domains from other membrane proteins may interfere with the *C*-terminus determined TGN-targeting events of CI-M6PR. On the other hand, without native membrane protein luminal/extracellular sequence, the *C*-terminal sequence of the receptor was sufficient for chimeric protein targeting to TGN.

### The HA tagged hCI-M6PR showed the similar membrane trafficking events as that of full-length CI-M6PR

**Fig.3:**
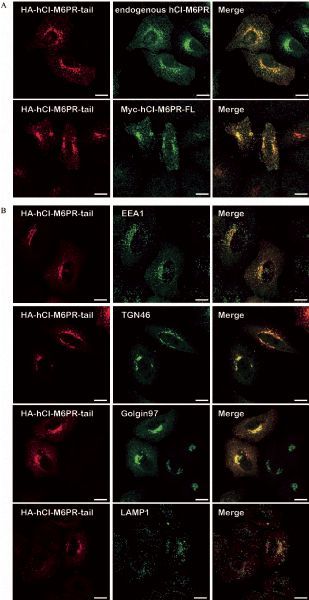
**The HA-tagged hCI-M6PR showed similar membrane trafficking events as that of full-length CI-M6PR. ** A: HA-hCI-M6PR-tail was transiently transfected in HeLa Swiss cells, followed by double immunofluorescent staining using anti-HA antibody and CI-M6PR specific antibody CD222 *(upper panel)*. Both HA-hCI-M6PR-tail and Myc-hCI-M6PR-FL were transiently transfected in HeLa Swiss cells, followed double immunofluorescent staining in HeLa Swiss cells using anti-HA antibody and anti-Myc antibody *(lower panel)*. Scale bar, 10 μm. B: HA-hCI-M6PR-tail was transiently transfected in HeLa Swiss cells, followed by double immunofluorescent staining using anti-HA antibody and antibodies for different subcellular organelle markers EEA1, Golgin97, TGN46, and LAMP1. Scale bar, 10 μm.

**Fig.4:**
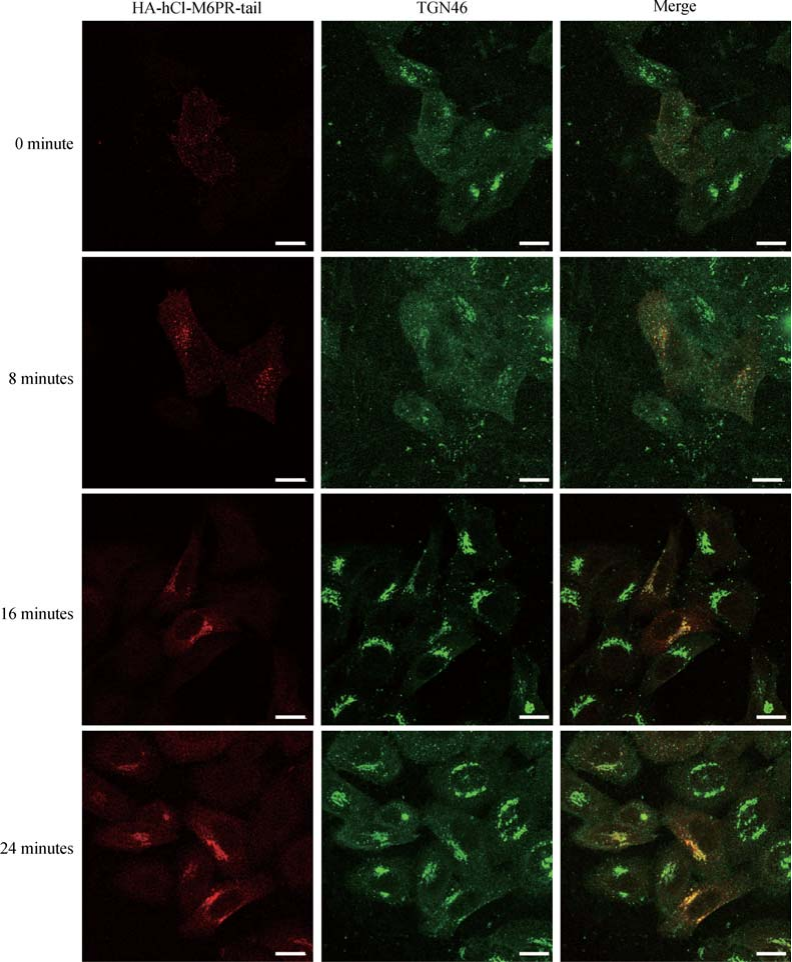
**The HA-tagged hCI-M6PR normally traffics between endosome, TGN and plasma membrane.** HeLa Swiss cells transiently transfected with HA-hCI-M6PR-tail were incubated with anti-HA antibodies, washed, and then warmed to 37 °C for 0, 8, 16, or 24 minutes before fixation. The cells were then labeled with antibodies against TGN46 followed by secondary antibodies. Scale bar, 10 μm.

As the luminal domain truncated chimeric protein (HA-hCI-M6PR-tail) showed sufficient TGN targeting pattern, we then examined whether this chimeric protein performed the same membrane trafficking events as that of the full-length hCI-M6PR. As shown in ***Fig. 3A***, immunostaining of the overexpressed HA-hCI-M6PR-tail showed significant co-localization with either endogenous hCI-M6PR which was marked by CD222, or overexpressed full-length Myc-hCI-M6PR (***Fig. 3A***). Next, we used different markers to verify the overall subcellular distribution of hCI-M6PR-tail. As shown in ***Fig. 3B***, overexpressed HA-hCI-M6PR-tail in HeLa Swiss cells significantly co-localized with both Golgi marker Golgin97 and TGN46 while only partially with early endosome maker EEA1, but rarely with lysosomal maker LAMP1, indicating efficient retrograde trafficking for the chimeric HA-hCI-M6PR-tail between endosome and TGN. To further demonstrate whether HA-hCI-M6PR-tail behaved the same as the full-length receptor for its membrane trafficking in HeLa Swiss cells^[19^‒^20]^, we performed an antibody uptake assay to examine the trafficking dynamics of the chimeric proteins *en route* to the TGN after internalization from the cell surface. As shown in ***Fig. 4***, after 24 minute chase at 37 °C, the majority of the anti-HA antibody labeled receptors were accumulated in the TGN region (***Fig. 4***), consistent with the trafficking routes and kinetics of the endogenous protein. These results indicated that the luminal domain truncated form of hCI-M6PR may serve as a new cargo model to investigate the mechanisms underlying the retrograde trafficking.

### The HA tagged form of hCI-M6PR underwent the retromer regulated membrane trafficking pathway

**Fig.5:**
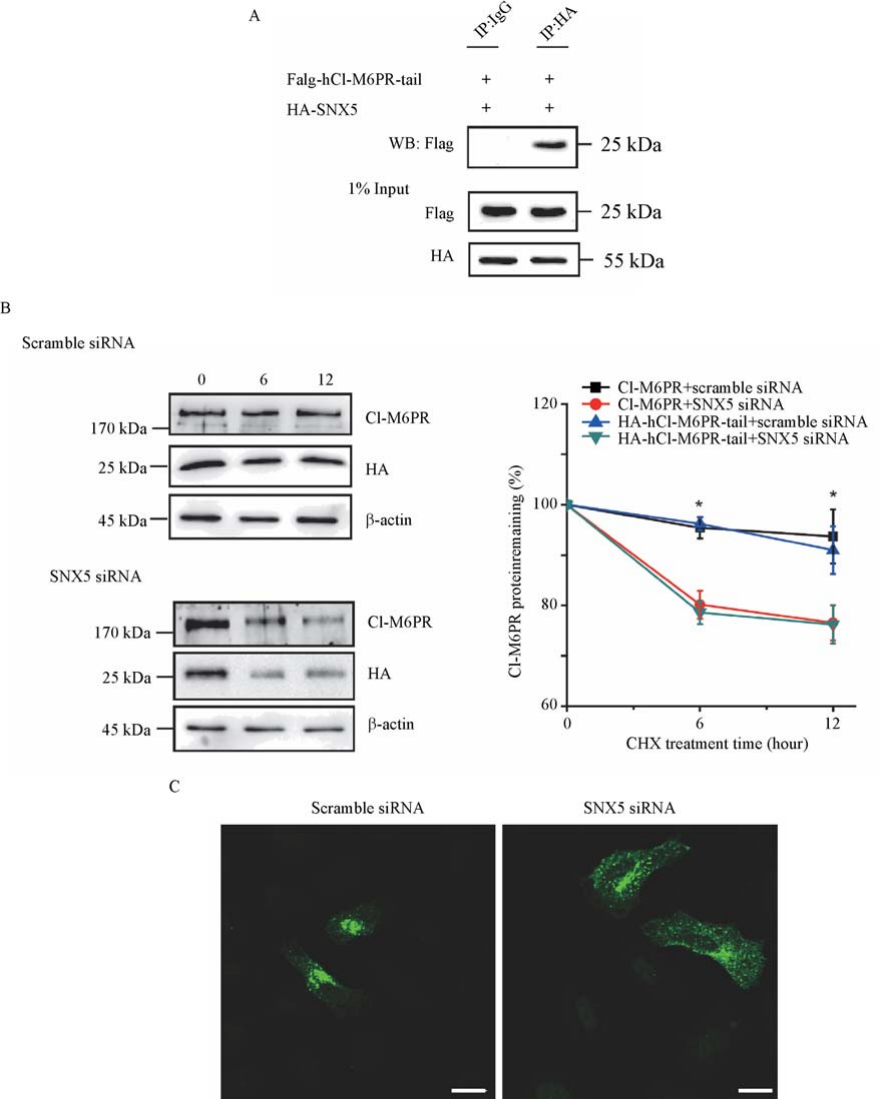
**The HA tagged hCI-M6PR underwent the retromer regulated membrane trafficking pathway.** A: Both HA-SNX5 and Flag-hCI-M6PR-tail were transiently overexpressed in COS7 cells, followed by cell lysates immunoprecipitated with anti-HA antibody and then analyzed with anti-Flag antibody using Western blotting. B: Half-life of HA-hCI-M6PR-tail expression was examined in control and SNX5-deleted HeLa Swiss cells after cycloheximide (CHX, 100 mg/mL) treatment on the indicated time points. HA-hCI-M6PR-tail expression level was detected with anti-HA antibody while endogenous CI-M6PR level was detected with anti-CD222 antibody by Western blot and quantified by densitometry analysis. The arbitrary densitometry value was measured using imaging analysis software Image J. Data (mean±SEM) were from the indicated number of independent experiments and comparisons were analyzed using one-way ANOVA followed by Tukey's *post hoc* test. *P < 0.05. C: The subcellular distribution of HA-hCI-M6PR-tail in control and SNX5-depleted HeLa Swiss cells was tested by immunofluorescent staining. Scale bar, 10 μm.

CI-M6PR has been considered as a classical retrograde trafficking cargo protein to be highly regulated by the retromer complex^[5^,^21^‒^22]^. Earlier studies suggested that VPS trimer (VPS26/29/35) of the retromer plays an important role in recognizing the cargo proteins through protein interaction^[3^,^12]^. However, two recent independent studies suggested that the SNX-BAR proteins rather than the VPS trimer of the retromer complex regulated the retrograde trafficking through direct binding to the *C*-terminus of CI-M6PR, specifically the WLM motif^[23^‒^24]^. To better understand whether the luminal truncated hCI-M6PR-tail protein was regulated by the retromer complex, we firstly examined whether SNX5 interacted with hCI-M6PR-tail. As shown in ***Fig. 5A***, the co-immunoprecipitation study on both Flag-hCI-M6PR-tail and HA-SNX5 overexpressed in COS7 cells revealed that SNX5 specifically interacted with hCI-M6PR-tail but not VPS35 (data not shown), supporting the latest understanding of a role of SNX5 in the membrane trafficking of the luminal truncated hCI-M6PR-tail. Furthermore, to determine whether SNX5 could functionally regulate the membrane trafficking of hCI-M6PR-tail, we performed half-life assay to examine its stability in HeLa Swiss cells. After blocking the protein synthesis, the expression level of hCI-M6PR-tail was stable over the course of 12 hours in scrambled siRNA treated control cells. In contrast, the expression levels of hCI-M6PR-tail were dramatically decreased by depleted SNX5 expression using specific SNX5 siRNA (***Fig. 5B***). Consistently, HA-hCI-M6PR-tail was localized at the periphery region subcellularly upon SNX5 depletion rather than in perinuclear TGN areas in control cells (***Fig. 5C***). Taken together, these data suggested that hCI-M6PR-tail contains sufficient TGN sorting signal to be regulated by the retromer complex through interacting with the SNX5 and thus can serve as the cargo protein for understanding molecular mechanisms underlying retrograde trafficking.

## Discussion

In this paper, we have reported that, by using chimeric protein strategy, TGN targeting of CI-M6PR may be influenced by luminal/extracellular domains. We also generated a chimeric protein of the receptor with triple HA tag to show its preferential targeting to TGN and interaction with SNX5, the key component of the retromer complex.

Although the stable transformant line in which the chimeric CD8-bCI-M6PR-C protein preferentially localized to TGN has been broadly used in the field^[12^‒^13]^, the similar constructs we used with both bovine and human receptors failed to confirm the TGN targeting in our transient overexpression system. Similar chimeric Tac-hCI-M6PR-C also consistently showed its mistargeting to non-TGN compartments (***Fig. 1E***), suggesting either a role of the luminal domain in regulating TGN targeting or insufficiency of *C*-terminus of CI-M6PR for TGN targeting. Interestingly, earlier studies indicated that the chimeric luminal domains from other irrelevant proteins might play a role in the subcellular distribution of the CI-M6PR. One such example was that the chimeric protein using the extracellular/luminal and transmembrane domains of epidermal growth factor receptor (EGFR) to fuse with the *C*-terminus of bovine CI-M6PR was mis-targeted to non-TGN compartments^[25]^. In addition, Waguri *et al.* indicated that the CI-M6PR luminal domain was required for tight interaction with endocytic compartments^[26]^. Importantly, the deletion mutant in our study without 1-13 repeating segments in the luminal domain of CI-M6PR also showed altered TGN localization (***Fig. 2B***), indicating that the disrupted luminal domain of the receptor may influence its targeting and trafficking.

One possibility of the luminal domain regulating the trafficking and targeting of the receptor is its dependence on dimerization related folding. Although under most circumstances the CI-M6PR behaves as a monomer, the protein is supposed to be a dimer as shown by its family member cation-dependent mannose 6-phophase receptor (CD-M6PR)^[27^‒^28]^. Brown *et al.* have suggested a model for the full-length luminal/extracellular domain in which even-numbered domains face one direction and odd-numbered domains face the opposite direction^[29]^. According to this model and well-known ligand binding sites, the even-numbered domains play a role in dimerization while the odd-numbered domains bind to ligands^[30^‒^31]^. Our deletion mutation which contains domain 14 and 15 is supposed to be able to form the dimer. However, this deletion mutation showed no preferential TGN localization, indicating that the dimerization of the CI-M6PR may not contribute to its targeting and trafficking. This result led us to consider another possibility of the role of ligand binding in the receptor’s targeting. Previous studies have suggested that the binding and dissociation of M6P-ligands triggered the translocation of CI-M6PR between intracellular compartments^[32^‒^33]^. Both the deletion mutation and chimeric proteins used in this study did not contain the binding sites for M6P and IGF2. Although we cannot rule out that ligand-unbound form of luminal sequence may contribute to their mis-targeting, our tagged chimeric protein strategy may provide some answers to this concern.

Regarding whether the *C*-terminus of CI-M6PR is sufficient for TGN targeting, we used triple HA tagged chimeric protein containing both TMD and *C*-terminus of CI-M6PR (HA-hCI-M6PR-tail) to test its TGN targeting. Our data has shown the chimeric protein with short non-native sequences localized preferentially to TGN, similar to the full length protein (***Fig. 1***). In addition, a GFP tagged chimeric receptor without luminal domain also showed typical TGN distribution pattern (data not shown) which is similar to the previous report^[26]^. Thus, these results strongly support the sufficiency of the C-terminus of CI-M6PR for its TGN targeting. Furthermore, our study indicated that SNX5, the key component of the retromer complex, regulated the trafficking of the tagged chimeric protein through interacting with the C-terminal domain, confirming the previous report that it was SNX5 rather than Vps35 interacting with the C-terminus of CI-M6PR to regulate the receptor’s retrograde trafficking^[23^‒^24]^. Thus, our study strongly suggested that only the innate full-length luminal domain of CI-M6PR contributes to its TGN-targeting while the partial deletion of luminal domain or an irrelevant luminal domain interfered with this event.

## References

[1] Ghosh P, Dahms NM, Kornfeld S. Mannose 6-phosphate receptors: new twists in the tale[J]. *Nat Rev Mol Cell Biol*, 2003, 4(3): 202‒212 . 12612639 10.1038/nrm1050

[2] Seaman MN. Cargo-selective endosomal sorting for retrieval to the Golgi requires retromer[J]. *J Cell Biol*, 2004, 165(1): 111‒122 . 15078902 10.1083/jcb.200312034 PMC2172078

[3] Arighi CN, Hartnell LM, Aguilar RC, Role of the mammalian retromer in sorting of the cation-independent mannose 6-phosphate receptor[J]. *J Cell Biol*, 2004, 165(1): 123‒133 . 15078903 10.1083/jcb.200312055 PMC2172094

[4] Hille-Rehfeld A. Mannose 6-phosphate receptors in sorting and transport of lysosomal enzymes[J]. *Biochim Biophys Acta*, 1995, 1241(2): 177‒194 . 7640295 10.1016/0304-4157(95)00004-b

[5] Wassmer T, Attar N, Bujny MV, A loss-of-function screen reveals SNX5 and SNX6 as potential components of the mammalian retromer[J]. *J Cell Sci*, 2007, 120(Pt 1): 45‒54 . 17148574 10.1242/jcs.03302

[6] Chen HJ, Remmler J, Delaney JC, Mutational analysis of the cation-independent mannose 6-phosphate/insulin-like growth factor Ⅱ receptor. A consensus casein kinase Ⅱ site followed by 2 leucines near the carboxyl terminus is important for intracellular targeting of lysosomal enzymes[J]. *J Biol Chem*, 1993, 268(30): 22338‒22346 . 8226743

[7] Wan L, Molloy SS, Thomas L, PACS-1 defines a novel gene family of cytosolic sorting proteins required for trans-Golgi network localization[J]. *Cell*, 1998, 94(2): 205‒216 . 9695949 10.1016/s0092-8674(00)81420-8

[8] Zhu Y, Doray B, Poussu A, Binding of GGA2 to the lysosomal enzyme sorting motif of the mannose 6-phosphate receptor[J]. *Science*, 2001, 292(5522): 1716‒1718 . 11387476 10.1126/science.1060896

[9] Doray B, Ghosh P, Griffith J, Cooperation of GGAs and AP-1 in packaging MPRs at the trans-Golgi network[J]. *Science*, 2002, 297(5587): 1700‒1703 . 12215646 10.1126/science.1075327

[10] Díaz E, Pfeffer SR. TIP47: a cargo selection device for mannose 6-phosphate receptor trafficking[J]. *Cell*, 1998, 93(3): 433‒443 . 9590177 10.1016/s0092-8674(00)81171-x

[11] Orsel JG, Sincock PM, Krise JP, Recognition of the 300-kDa mannose 6-phosphate receptor cytoplasmic domain by 47-kDa tail-interacting protein[J]. *Proc Natl Acad Sci U S A*, 2000, 97(16): 9047‒9051 . 10908666 10.1073/pnas.160251397 PMC16819

[12] Seaman MNJ. Cargo-selective endosomal sorting for retrieval to the Golgi requires retromer[J]. *J Cell Biol*, 2004, 165(1): 111‒122 . 15078902 10.1083/jcb.200312034 PMC2172078

[13] Niu Y, Zhang C, Sun Z, PtdIns(4)P regulates retromer-motor interaction to facilitate dynein-cargo dissociation at the trans-Golgi network[J]. *Nat Cell Biol*, 2013, 15(4): 417‒429 . 23524952 10.1038/ncb2710

[14] Navarro Negredo P, Edgar JR, Manna PT, The WDR11 complex facilitates the tethering of AP-1-derived vesicles[J]. *Nat Commun*, 2018, 9(1): 596 . 29426865 10.1038/s41467-018-02919-4 PMC5807400

[15] Hancock MK, Haskins DJ, Sun G, Identification of residues essential for carbohydrate recognition by the insulin-like growth factor Ⅱ/mannose 6-phosphate receptor[J]. *J Biol Chem*, 2002, 277(13): 11255‒11264 . 11799115 10.1074/jbc.M109855200

[16] Schmidt B, Kiecke-Siemsen C, Waheed A, Localization of the insulin-like growth factor Ⅱ binding site to amino acids 1508-1566 in repeat 11 of the mannose 6-phosphate/insulin-like growth factor Ⅱ receptor[J]. *J Biol Chem*, 1995, 270(25): 14975‒14982 . 7797478 10.1074/jbc.270.25.14975

[17] Colgan L, Liu H, Huang SY, Dileucine motif is sufficient for internalization and synaptic vesicle targeting of vesicular acetylcholine transporter[J]. *Traffic*, 2007, 8(5): 512‒522 . 17451554 10.1111/j.1600-0854.2007.00555.x

[18] Breusegem SY, Seaman MN. Image-based and biochemical assays to investigate endosomal protein sorting[J]. *Methods Enzymol*, 2014, 534: 155‒178 . 24359953 10.1016/B978-0-12-397926-1.00009-3

[19] Dell’Angelica EC, Payne GS. Intracellular cycling of lysosomal enzyme receptors: cytoplasmic tails’ tales[J]. *Cell*, 2001, 106(4): 395‒398 . 11525725 10.1016/s0092-8674(01)00470-6

[20] Rohn WM, Rouillé Y, Waguri S, Bi-directional trafficking between the trans-Golgi network and the endosomal/lysosomal system[J]. *J Cell Sci*, 2000, 113(Pt 12): 2093‒2101 . 10825282 10.1242/jcs.113.12.2093

[21] Rojas R, Kametaka S, Haft CR, Interchangeable but essential functions of SNX1 and SNX2 in the association of retromer with endosomes and the trafficking of mannose 6-phosphate receptors[J]. *Mol Cell Biol*, 2007, 27(3): 1112‒1124 . 17101778 10.1128/MCB.00156-06 PMC1800681

[22] Mari M, Bujny MV, Zeuschner D, SNX1 defines an early endosomal recycling exit for sortilin and mannose 6-phosphate receptors[J]. *Traffic*, 2008, 9(3): 380‒393 . 18088323 10.1111/j.1600-0854.2007.00686.x

[23] Kvainickas A, Jimenez-Orgaz A, Nägele H, Cargo-selective SNX-BAR proteins mediate retromer trimer independent retrograde transport[J]. *J Cell Biol*, 2017, 216(11): 3677‒3693 . 28935632 10.1083/jcb.201702137 PMC5674888

[24] Simonetti B, Danson CM, Heesom KJ, Sequence-dependent cargo recognition by SNX-BARs mediates retromer-independent transport of CI-MPR[J]. *J Cell Biol*, 2017, 216(11): 3695‒3712 . 28935633 10.1083/jcb.201703015 PMC5674890

[25] Dintzis SM, Pfeffer SR. The mannose 6-phosphate receptor cytoplasmic domain is not sufficient to alter the cellular distribution of a chimeric EGF receptor[J]. *EMBO J*, 1990, 9(1): 77‒84 . 2153081 10.1002/j.1460-2075.1990.tb08082.x PMC551632

[26] Waguri S, Tomiyama Y, Ikeda H, The luminal domain participates in the endosomal trafficking of the cation-independent mannose 6-phosphate receptor[J]. *Exp Cell Res*, 2006, 312(20): 4090‒4107 . 17069798 10.1016/j.yexcr.2006.09.024

[27] Byrd JC, Park JH, Schaffer BS, Dimerization of the insulin-like growth factor Ⅱ/mannose 6-phosphate receptor[J]. *J Biol Chem*, 2000, 275(25): 18647‒18656 . 10764761 10.1074/jbc.M001273200

[28] Byrd JC, MacDonald RG. Mechanisms for high affinity mannose 6-phosphate ligand binding to the insulin-like growth factor Ⅱ/mannose 6-phosphate receptor[J]. *J Biol Chem*, 2000, 275(25): 18638‒18646 . 10764735 10.1074/jbc.M000010200

[29] Brown J, Esnouf RM, Jones MA, Structure of a functional IGF2R fragment determined from the anomalous scattering of sulfur[J]. *EMBO J*, 2002, 21(5): 1054‒1062 . 11867533 10.1093/emboj/21.5.1054 PMC125895

[30] Dahms NM, Rose PA, Molkentin JD, The bovine mannose 6-phosphate/insulin-like growth factor Ⅱ receptor. The role of arginine residues in mannose 6-phosphate binding[J]. *J Biol Chem*, 1993, 268(8): 5457‒5463 . 8449908

[31] Garmroudi F, Devi G, Slentz DH, Truncated forms of the insulin-like growth factor Ⅱ (IGF-Ⅱ)/mannose 6-phosphate receptor encompassing the IGF-Ⅱ binding site: characterization of a point mutation that abolishes IGF-Ⅱ binding[J]. *Mol Endocrinol*, 1996, 10(6): 642‒651 . 8776724 10.1210/mend.10.6.8776724

[32] Brown WJ, Constantinescu E, Farquhar MG. Redistribution of mannose-6-phosphate receptors induced by tunicamycin and chloroquine[J]. *J Cell Biol*, 1984, 99(1 Pt 1): 320‒326 . 6330128 10.1083/jcb.99.1.320 PMC2275644

[33] Brown WJ, Farquhar MG. Accumulation of coated vesicles bearing mannose 6-phosphate receptors for lysosomal enzymes in the Golgi region of I-cell fibroblasts[J]. *Proc Natl Acad Sci U S A*, 1984, 81(16): 5135‒5139 . 6147848 10.1073/pnas.81.16.5135 PMC391652

